# A MIXED-METHODS EVALUATION OF A 20-YEAR SENIOR MENTOR PROGRAM FOR MEDICAL STUDENTS

**DOI:** 10.1093/geroni/igz038.2464

**Published:** 2019-11-08

**Authors:** Tina R Kilaberia, Edward Ratner, June Englund

**Affiliations:** 1 Minnesota State University, Mankato, Mankato, Minnesota, United States; 2 University of Minnesota - Twin Cities, Minneapolis, Minnesota, United States; 3 Augustana Care Organization, Minneapolis, Minnesota, United States

## Abstract

The purpose of this mixed-methods evaluation is to demonstrate the success from the past 20 class years of continuous use of senior mentors to teach geriatrics to approximately 3400 Year 1 and Year 2 medical students in a home visit model. Using a pre-test post-test design (N=131), we evaluated student satisfaction, self-confidence in various geriatrics assessments, and performance on an Objective Structured Clinical Evaluation (OSCE). We additionally conducted a formal qualitative evaluation of students’ open-ended reflections (N=97) across three years, from 2016-18, on what they learned. In addition, relying on the principles of community-based research, we collaborated with a volunteer senior on all aspects of this evaluation, and included a community of seniors at a large urban senior care high-rise to enhance our interpretive validity by asking seniors to reflect on a set of representative quotes for each qualitative theme. The senior mentor home visit model demonstrated high satisfaction, improved self-confidence in geriatric assessment, attainment of competencies, and improvement in attitude scores. With regard to the qualitative portion of the evaluation, six themes about learning were recognized: (1) how to perform an interview and exam in a home setting, (2) how life for seniors is different than students expected, (3) the value of physical infrastructure and amenities in senior housing, (4) the importance of senior’s community, including family, neighbors, spiritual community, (5) challenges with aging, and (6) strengths among seniors in coping with such challenges while maintaining individual agency.

